# Supplementary material for: “The only friend I had was my gun”: A mixed-methods study of gun culture in school shootings
Source: PLoS One. 2025 Apr 23;20(4):e0322195. doi: 10.1371/journal.pone.0322195 (PMC12017492; doi:10.1371/journal.pone.0322195)
Supplement: S1 Data — Cited data sources. This supporting information lists all sources referenced in the article. The list does not include all analyzed sources for this project, as these by far exceed those listed here. It includes sources that are directly referenced in the article when discussing cases as examples of larger identified patterns. (PDF) [file pone.0322195.s001.docx]

Supporting Information

**“The only friend I had was my gun”: A mixed-methods study of gun culture in school shootings**

**S1. Cited Data Sources.**

This list does not include all analyzed sources for this project, as these by far exceed those listed here. The list includes sources that are directly referenced in the article when discussing cases as examples of larger identified patterns.

[D1] Interview with shooter's father, in: Bragg, Rick, Dirk Johnson, John Kifner, and Sam Howe Verhovek. 1998. “From Wild Talk and Friendship To Five Deaths in a Schoolyard”. *The New York Times*. March 29. Available at: https://www.nytimes.com/1998/03/29/us/from-wild-talk-and-friendship-to-five-deaths-in-a-schoolyard.html

[D2] Interview with teacher, reported in: Harding, David J., Cybelle Fox, and Jal D. Mehta. 2002. “Studying Rare Events through Qualitative Case Studies: Lessons from a Study of Rampage School Shootings.” *Sociological Methods & Research* 31(2):174–217. doi: 10.1177/0049124102031002003.

[D3] Family Picture of Perpetrator, printed on the cover of *Time Magazine*, April 06, 1998. For the original picture see: Miller, Jacob. 2017. “24 Images of the Heartbreaking Jonesboro Middle School Massacre.” *HistoryCollection.Com*. Available at: https://historycollection.com/24-images-heartbreaking-jonesboro-middle-school-massacre/

[D4] Family Picture of Perpetrator, printed among other places in: Cox JW. Inside an accused school shooter’s mind: A plot to kill ‘50 or 60. If I get lucky maybe 150.’ Washington Post. 2018 Mar 3 [cited 2021 Nov 17]; Available from: https://www.washingtonpost.com/local/inside-a-teen-school-shooters-mind-a-plot-to-kill-50-or-60-if-i-get-lucky-maybe-150/2018/03/03/68cc673c-1b27-11e8-ae5a-16e60e4605f3_story.html

[D5] Statement by the Perpetrator, reported in: Anderson Country Sheriff’s Office. 2016. *Transcript of Record - Interview with Jesse Osborne*. Anderson, South Carolina: State of South Carolina, County of Anderson in the Family Court. Available among other places at: https://www.scribd.com/document/371434649/Jesse-Osborne-transcript

[D6] FBI Report: Anchorage Office of the FBI. 1966. *Whitness Statement Report*. Anchorage: Collections of the Austin History Center.

[D7] Among other sources reported in: Wallenfeldt, Jeff. 2022. “Texas Tower Shooting of 1966.” *Britannica*.

[D8] Family picture, printed among other places in: *LIFE Magazine*. 1966. “The Texas Sniper.” *Life Magazines*, December 8. https://commons.wikimedia.org/wiki/File:Charles_Whitman_1943.jpg

[D9] Statement by Perpetrator, reported in: Larson, Eric. 1993. “The Story of a Gun.” *The Atlantic*, January 1993 issue. Available at: https://www.theatlantic.com/magazine/archive/1993/01/the-story-of-a-gun/303531/

[D10] Statement by Psychologist, reported in: M. L. Sullivan and R. T. Guerette, “The Copycat Factor: Mental Illness, Guns, and the Shooting Incident at Heritage High School, Rockdale County, Georgia,” in *Deadly Lessons: Understanding Lethal School Violence*, National Research Council, Ed., Washington, D.C.: The National Academies Press, 2003, pp. 25–69. Available at: https://doi.org/10.17226/10370

[D11] R. Bragg, D. Johnson, J. Kifner, S. H. Verhovek, and W. B. M. Kifner, “From Wild Talk and Friendship To Five Deaths in a Schoolyard,” The New York Times, Mar. 29, 1998. Accessed: Aug. 15, 2024. Available at: https://www.nytimes.com/1998/03/29/us/from-wild-talk-and-friendship-to-five-deaths-in-a-schoolyard.html

[D12] Statement by JROTC teammate, reported in: Wright, Mike and Associated Press. 2018. “Florida Shooting Suspect Nikolas Cruz Was Member of School’s Rifle Team and Described as a ‘Very Good Shot.’” *The Telegraph*, February 17. Available at: https://www.telegraph.co.uk/news/2018/02/17/florida-shooting-suspect-nikolas-cruz-member-schools-rifle-team/

[D13] Statement by neighbour, reported in: Healy, Jack, and Ian Lovett. 2015. “Oregon Killer Described as Man of Few Words, Except on Topic of Guns.” *The New York Times*, February 10. Available at: https://www.nytimes.com/2015/10/03/us/chris-harper-mercer-umpqua-community-college-shooting.html

[D14] Perpetrator statement in interrogation, see: WBAL Radio, *Robert Gladden Jr Questioned by Baltimore County Police Part 1*, (Feb. 26, 2013). Accessed: Jan. 28, 2022. [Video]. Available at: https://www.youtube.com/watch?v=_lJjmiSC9Bs

[D15] Perpetrator statements, cited among other places in: J. Schulberg, “Kip Kinkel Is Ready To Speak,” *HuffPost*, Jun. 12, 2021. Accessed: Jun. 17, 2022. Available at: https://www.huffpost.com/entry/kip-kinkel-is-ready-to-speak_n_60abd623e4b0a2568315c62d

[D16] Statements by various friends, reported in: T. Clouse, C. Sokol, and E. Francovich, “Signs and warnings failed to prevent Freeman school shooting | The Spokesman-Review,” *The Spokesman-Review*, Sep. 15, 2017. Accessed: Jan. 28, 2022. Available: at https://www.spokesman.com/stories/2017/sep/15/signs-and-warnings-failed-to-prevent-freeman-schoo/

[D17] Prosecutor statement, reported in: M. Alesia, E. K. Fittes, C. Lanich, V. Ryckaert, and R. Martin, “Noblesville shooting victim’s parents speak: ‘Maximum sentence’ needed for teen suspect,” *IndyStar*, 2018. Accessed: Jan. 28, 2022. Available at: https://www.indystar.com/story/news/local/hamilton-county/education/2018/11/05/noblesville-west-middle-school-shooting-motive-ella-whistler-jason-seaman/1823155002/

[D18] Notes by shooter's psychologist, reported in: PBS, “Who Is Kip Kinkel? - Chronology | The Killer At Thurston High,” PBS Frontline. Accessed: Jan. 28, 2022.. Available at: https://www.pbs.org/wgbh/pages/frontline/shows/kinkel/kip/cron.html

[D19] Statements made in court records, reported in: W. B. Patrick, “An Incident of School Violence in East Greenbush, New York,” 2013. Available at: https://www.yumpu.com/en/document/view/4523575/an-incident-of-school-violence-in-east-william-b-patrick

[D20] Neighbor's statement, reported in: KCAL News, “Neighbor Describes Saugus High School Shooting Suspect Nathaniel Berhow’s Family As ‘Good People,’” 2020. Available at: https://www.youtube.com/watch?v=7Z-u57Z0TM8

[D21] Psychologist statement, reported in: M. L. Sullivan and R. T. Guerette, “The Copycat Factor: Mental Illness, Guns, and the Shooting Incident at Heritage High School, Rockdale County, Georgia,” in *Deadly Lessons: Understanding Lethal School Violence*, National Research Council, Ed., Washington, D.C.: The National Academies Press, 2003, pp. 25–69. Available at: https://doi.org/10.17226/10370

[D22] Court testimony, reported in: W. Glaberson, “Man and His Son’s Slayer Unite to Ask Why,” *The New York Times*, Dec. 04, 2000. Available at: https://www.nytimes.com/2000/04/12/us/man-and-his-son-s-slayer-unite-to-ask-why.html

[D23] Reported in: D. Vann, “Portrait of the School Shooter as a Young Man,” Esquire, August 2008. Accessed: Mar. 20, 2022. Available at: https://classic.esquire.com/article/2008/8/1/portrait-of-the-school-shooter-as-a-young-man

[D24] Virginia Tech Review Panel, “Mass Shootings at Virginia Tech, April 16, 2007,” Virginia, Aug. 2007. Available at the Library of Congress: https://catalog.loc.gov/vwebv/search?searchCode=LCCN&searchArg=2008397165&searchType=1&permalink=y

[D25] Interrogation Video with perpetator, available among other places at: Sun Sentinel, *FULL VIDEO: Nikolas Cruz interrogation after Parkland school shooting*, (Aug. 09, 2018). Accessed: Nov. 18, 2021. Available at: https://www.youtube.com/watch?v=ob77T58HeIo

[D26] Office of the State’s Attorney Judicial District of Danbury, “Report of the State’s Attorney for the Judicial District of Danbury on the Shootings at Sandy Hook Elementary School and 36 Yogananda Street, Newtown, Connecticut on December 14, 2012,” Danbury, Nov. 2013. Available at CT State Library: https://cslib.contentdm.oclc.org/digital/collection/p128501coll2/id/262076

[D27] North Carolina Court of Appeals, “Appeal State of North Carolina v. Alvaro Rafael Castillo,” North Carolina Court of Appeals, Jul. 2009. Available among other places at: https://caselaw.findlaw.com/court/nc-court-of-appeals/1574906.html

[D28] Jefferson County Sherrif’s Office, “Jefferson County Sherrif’s Office Final Report on the Columbine High School Shooting,” Jefferson County, Colorado, May 2000. Available at: http://edition.cnn.com/SPECIALS/2000/columbine.cd/Pages/TOC.htm

[D29] Police statements, reported in: S. Dance and E. Cox, “Police: Perry Hall shooting suspect knew of stepfather’s gun cache,” *The Baltimore Sun*, Aug. 31, 2012. Available at: https://www.baltimoresun.com/latest/bs-md-perry-hall-shooting-20120831-story.html
