# Supplementary material for: “The only friend I had was my gun”: A mixed-methods study of gun culture in school shootings
Source: PLoS One. 2025 Apr 23;20(4):e0322195. doi: 10.1371/journal.pone.0322195 (PMC12017492; doi:10.1371/journal.pone.0322195)
Supplement: S2 Data — Full dataset. This table summarizes key data points from the analysis in numerical form and provides information from the in-depth case analyses. Whether gun access was easy or not was coded as fuzzy sets: very easy = 1; easy = 0.7; difficult = 0.3; very difficult =0. Access of the gun was coded as 1 = from home, 2 = legally bought, 3 = other. The notes provide a brief summary on gun culture, access, and relevance of the gun in the shooters’ life for each case. (PDF) [file pone.0322195.s002.docx]

Supporting Information

**“The only friend I had was my gun”: A mixed-methods study of gun culture in school shootings**

**S2. Table. Full Dataset.**

This table summarizes key data points from the analysis in numerical form and provides information from the in-depth case analyses. Whether gun access was easy or not was coded as fuzzy sets: very easy= 1; easy = 0.7; difficult= 0.3; very difficult=0. Access of the gun was coded as 1= from home, 2= legally bought, 3= other. The notes provide a brief summary on gun culture, access, and relevance of the gun in the shooters’ life.

| **Shooting date** | **Shooter age** | **Gun access** | **Gun origin** | **Notes** |
| --- | --- | --- | --- | --- |
| 08/66 | 25 | 1 | 2 | Shooter shot firearms from young age and shooting was important to father. Shooter owned a number of firearms legally. |
| 09/71 | 14 | NA | NA | Overall lack of data on case. |
| 11/71 | 23 | 1 | 2 | Shooter was an army veteran with legal access to firearms. |
| 12/74 | 17 | 1 | 2 | Shooter had several guns prominently displayed at home and was part of the school’s rifle team. |
| 10/79 | 19 | 1 | 2 | Shooter was a gun enthusiast and with legal access to firearms. |
| 01/85 | 14 | 1 | 1 | Shooter was a gun enthusiast and father collected firearms as a hobby. Shooter took guns from his father. |
| 02/87 | 17 | 1 | 3 | Shooter obtained the gun from the biology teacher, who was the keeper of the school guns. Shooter told the teacher he had forgotten to unload the gun earlier in practice. The teacher let him into the gun storage and gave him the gun. |
| 12/88 | 16 | 0.7 | 3 | Shooter convinced an older cousin to buy the firearm for him and convinced him to drive to a bigger gun store that does not require a waiting period. Shooter found solace in guns and everyone at school knew about his obsession with firearms. |
| 01/89 | 24 | 0.7 | 2 | Shooter bought all of his guns with ease, despite being repeatedly in trouble with the law and getting multiple mental health treatments for suicidal thoughts and schizophrenia. He lied and gave a false information on the federal form required for the purchase and was given the firearms. |
| 05/92 | 20 | 1 | 2 | Shooter showed a strong gun fascination and spend a lot of time shooting for leisure. His psychiatrist claimed he bought firearms due to struggles with his masculinity. He purchased a number of guns legally from various shops. |
| 12/92 | 18 | 1 | 2 | Bought the gun legally after turning 18 without a waiting period and had ammunition delivered to his campus. The gun store helped him make the gun purchase and advised him how to avoid the waiting period. The seamless order convinced him that his attack was guided by a “divine force”. |
| 01/93 | 17 | 1 | 1 | Took the gun from the family home. The gun belonged to his father. He hunted with his father and uncle before, but was himself more into books. |
| 10/95 | 16 | 0.7 | 3 | Had the firearm at home. He had found it in a car one year prior during a break-in. |
| 11/95 | 17 | 1 | 1 | Father bought the gun for the shooter for his birthday and had it registered to himself. Shooter was fascinated with guns and violent movies. |
| 02/96 | 14 | 1 | 1 | Hunting and target shooting were central recreational activities  for the family. The shooter learned how to shoot from his father, who had a gun arsenal. The shooter had free access to this arsenal and took two guns from the father and one from the mother. |
| 02/96 | 16 | 1 | 3 | Shooter wanted to get a firearm after a breakup. Shooter talked to several people regarding his relationship problems and desire to get a gun. He then borrowed the gun from a friend the night before. |
| 10/97 | 16 | 1 | 1 | Shooter went hunting, among others with a neighbor, father and brother who owned guns for hunting. He had easy access to the guns at home. |
| 12/97 | 14 | 0.7 | 1 | Shooter was from a hunting community and learned to shoot at summer camp. He stole his father’s gun several weeks before the shooting, then climbed through the window of a friends’ house to get the key to the gun safe and ammunition. He stole a total of nice firearms from his father and neighbor over the period of two months and hid them behind Lego boxes in his room. |
| 12/97 | 14 | 1 | 1 | Family had a gun at home that the shooter had access to. The gun belonged to his father. |
| 03/98 | 11 | 0.7 | 1 | Shooter was from an avid hunting family; his parents ran a local gun club and he was given his first gun at age 6. At age 11 he won awards for his marksman ship and was fascinated with firearms. Together with his partner (row below) they took three unsecured guns from his home and several firearms from his grandfather’s home. |
| 03/98 | 13 | 0.7 | 1 | Because shooter’s stepfather had a criminal record, he did not have any weapons in his house. All weapons were obtained through his partner’s family (see above). |
| 04/98 | 14 | 1 | 1 | Shooter was from a hunting community and went shooting with friends. The father had kept his gun in a dresser drawer and when the shooter was alone at home one day, he went through parent’s belongings and found it. |
| 05/98 | 15 | 0.7 | 1 | Shooter showed strong interest in guns and explosives. His father bought him several weapons for showing apparent mental health improvement. This was against the psychologists’ recommendation. The shooter claimed the guns were important to him feeling safe and secure and being able to take control. |
| 04/99 | 15 | 1 | 1 | Shooter took his grandfather’s shotgun and brought it to school on the school bus, wrapped in a blanket. |
| 04/99 | 18 | 0.7 | 3 | Shooter was fascinated with guns and explosives and practiced shooting at a shooting range. He bought guns with his partner (row below) through a friend who could obtain them legally. |
| 04/99 | 17 | 0.7 | 3 | Never showed a fascination with guns, but his school shooting partner did (see above). They practiced shooting the gun together at a gun range. |
| 05/99 | 15 | 1 | 1 | Shooter had easy access to the large collection of guns owned by stepfather. He showed a strong firearm fascination and started using them at age 7. He regularly went target shooting and hunting with his mother. Friends say he constantly talked about guns with enthusiasm. The psychologist who interviewed him on retainer from his defence counsel, later testified that “guns were the love of his life”. |
| 11/99 | 12 | 1 | 1 | Shooter took the gun from home. |
| 12/99 | 13 | 1 | 1 | Shooter used his father’s gun. |
| 03/01 | 15 | 1 | 1 | Guns were prominent in the family. Shooter took his father’s gun from home. |
| 03/01 | 18 | 1 | 1 | Shooter took the guns from home, where he lived with his mother. It is unclear who bought them. |
| 01/02 | 43 | 1 | 2 | Shooter bought the firearm several months prior to the shooting. |
| 05/03 | 62 | 0.7 | 2 | Bought his firearms, but data did not specify when and where. |
| 02/04 | 16 | 1 | 1 | Since shooter was planning a hunting trip with his half-brother in a few months, his mother bought him a gun and went to practice with him. She asked his psychiatrist who saw it as a good opportunity for bonding. Shooter committed the shooting 48 hours after he was gifted his own gun. |
| 03/05 | 16 | 0.7 | 1 | Shooter was from an avid hunting family. Grandfather and cousin went hunting with him. The grandfather owned many guns and kept one “around for the kids for hunting”. Shooter had the rifle at least a year before the shooting. After he shoot his grandfather with the weapon, he also took grandfather’s pistol and shotgun, which he used for the attack. |
| 03/06 | 14 | 1 | 1 | No hunting background. His father, whom the shooter described as abusive, owned the gun. Father had given him three bullets the night before, he used these three bullets in the shooting. |
| 08/06 | 18 | 1 | 2 | Shooter owned guns legally. He bought one of his guns five days after he was released from an involuntary stay at a mental health clinic for depression and psychosis. There he was advised to have no contact with guns. Shooter gained a lot of comfort and courage from his guns, stating: “Guns are just like toys and lovers for me. I adore them. They are fun to play with. I love my weapons.” |
| 09/06 | 15 | 0.7 | 1 | Abusive father had guns in the bedroom, likely for self-defense reasons. Shooter went into his parents’ bedroom, which he was not allowed to, and took two guns from the father. |
| 10/06 | 13 | 1 | 1 | The family had several guns and said many of the guns were registered in the mother’s name and were acquired as hand-me-downs from relatives. Father was prohibited the possession of firearms, but said he thought this meant he was not supposed to be found carrying or buying weapons. Shooter obtained gun from his father’s safe, the key was in an ashtray with other keys. |
| 04/07 | 23 | 1 | 2 | Shooter purchased two guns (both semiautomatic), one of them across the street from his university. He was able to buy a gun although he had a known mental illness and was involuntarily admitted to a hospital for being a risk to himself and others. But since he registered for out-patient treatment he was never registered to the database. He went to a shooting range to practice. |
| 10/07 | 14 | 1 | 1 | Shooter likely got firearms from his brother, who was earlier incarcerated for weapons charges. |
| 12/07 | 24 | 1 | 2 | Shooter was from a hunting area. He legally acquired an arsenal of firearms in the months before the shooting. He told his mother that he was going to have deliveries of ammunition to their house for hunting purposes. The amount of ammunition was so high that the dealers and his father grew concerned. |
| 02/08 | 23 | 1 | 2 | Bought the revolver and ammunition from a pawn shop shortly before the shooting. |
| 02/08 | 27 | 1 | 2 | Learned how to shoot at a correctional facility where he worked before. Legally bought several firearms though an online provider, despite earlier mental health treatment. He committed the shooting two days after the guns arrived. |
| 04/09 | 18 | 1 | 1 | He took three firearms from his father’s place. |
| 05/09 | 15 | 1 | 1 | Shooter took his father’s gun and later bemoaned that he did not find more bullets at home to be able to fire at police. |
| 02/10 | 32 | 1 | 1 | From a hunting family. He used his father’s hunting rifle that was stored behind a door. |
| 02/12 | 17 | 1 | 1 | Shooter took the gun from his uncle’s room at his grandparent’s place, where it was stored under a desk. He found ammunition on the other side of the same room. |
| 04/12 | 43 | 1 | 2 | Had bought his firearm legally earlier in the year. |
| 08/12 | 15 | 1 | 1 | Had been shooting guns since he was six. Found the gun and bullets at his parent’s house when they forgot it in the basement. Usually, the guns were locked in a safe. |
| 12/12 | 20 | 1 | 1 | Shooter went shooting with his mother as a bonding experience. His mother bought him several firearms due to his fascination with them since years before the attack. The firearms were stored in a vault but the shooter knew the combination. |
| 04/13 | 18 | 1 | 2 | Shooter bought his gun legally from a licensed firearm dealer. He learned how to shoot in a 12-week citizens police academy course. Shooter later said that he planned a shooting earlier at his high school but had no gun access then and therefore did not do it. |
| 10/13 | 12 | 1 | 1 | Physically abusive father had the gun at home because he admired guns. The gun was a gift from a client, because shooter’s father could not afford one. The client had bought the gun from someone who had inherited it from his father-in-law. Shooters’ family had two guns in the house, one of which was locked away, one was hidden in a box on top of the refrigerator. Shooter found the latter and used it in the shooting. |
| 12/13 | 18 | 1 | 2 | Shooter purchased the firearm legally in a store. He passed the background check despite being in mental health treatment for impulse-control issues and anger issues, as well as being on medication at the time. |
| 01/14 | 12 | 1 | 1 | Father and son went hunting together. Shooter obtained the gun from his father, who had several unlocked firearms in the house, with the one used for the shooting not being locked. |
| 06/14 | 15 | 1 | 1 | Shooter was enthusiastic about guns. He was most engaged talking to others, when the topic was firearms. He loved hunting and was in the JROTC. His brother and father both had guns, who they say they were locked away, but the key to the safe was on their house key chain in the kitchen and the shooter seemingly took the keys from there to open the safe. |
| 11/14 | 31 | 1 | 2 | Shooter purchased the gun legally, in his own mind for self-defense reasons, as he was suffering from severe schizophrenia. |
| 10/15 | 26 | 1 | 2 | Shooter loved guns. They were one of the few things he liked. He went practice shooting a lot and bonded with his mother at shooting ranges. Firearms were the subject that got the otherwise quiet shooter to talk. He was in military training for a month, where he learned to shoot. All 14 weapons were legally purchased. |
| 02/16 | 14 | 1 | 1 | Gun town and strong history of guns in the family; shooter’s grandfather was avid hunter and NRA advocate and his grandmother bought him a BB gun for his birthday. The night before the shooting, he talked with his great-grandmother about hunting. He showed off his BB gun to her and she showed him her handgun, which he later took home and used for the shooting the next day. |
| 04./16 | 18 | 1 | 2 | Shooter went practice shooting from a young age. Target practice and airsoft battles were shared by many boys in the community and he was a member of a group that stalked one another as snipers. His mother allowed him to buy a gun at a gun show two weeks after his breakup, since he was depressed and bullied but recently feeling better. Two weeks later he committed the shooting. |
| 09/16 | 14 | 1 | 1 | Shooter grew up in a gun-loving family around big guns from a young age. His father had many guns, several of which were locked away. Shooter took a gun that he found in a dresser drawer and committed the shooting. |
| 01/17 | 17 | 1 | 1 | Shooter’s shotgun was a Christmas gift from his grandparents. The gun had had been kept in his mother’s room because he had once admitted to shooting and killing a cat. But he had access to it in her room. |
| 09/17 | 15 | 1 | 1 | Shooter was fascinated with guns. Father had the gun for self-defense, as they had moved to a neighborhood in the city and the father was worried about safety there. Shooter knew the code to his father’s safe and bragged about it to friends. When he was expelled (for threatening to shoot up the school), his father was informed and asked to change the code, but apparently did not do so. |
| 09/17 | 14 | 1.0 | 1 | Took the gun from his father’s safe. Data does not specify how but it does not seem likely that he would have been able to get it without a key, or the safe being unlocked. |
| 12/17 | 21 | 1 | 2 | Shooter legally bought gun a month before the shooting, as well as ammunition. The police had investigated him before, after he asked in a gaming forum where he could buy a cheap assault rifle for a mass shooting. But since he had no criminal history, he could still purchase the firearm legally. When making his purchase his father joked that he should not use it for a school shooting. |
| 01/18 | 15 | 1 | 1 | Step-father likely had the gun for self-defense. Shooter got his stepfather’s gun from the shelf in the bedroom closet and smuggled it out with some laundry. |
| 02/18 | 19 | 1 | 2 | Shooter was very passionate about firearms, which were his only hobby. He was in the JROTC and a good shot. Shooter legally bought firearms, despite severe mental disorders and being previously investigated by the FBI for planning a shooting. |
| 04/18 | 19 | 1 | 2 | Shooter bought the gun legally, although being Baker Acted, which would prohibit a gun purchase. However, he went to an online site and got his firearm about a week later from a private seller without paperwork or a background check. He found out about this option to acquire a gun from a news report on a school shooting two months earlier (see row above). |
| 05/18 | 19 | 1 | 1 | Shooter took his mother’s gun from home. His mother purchased it in 2012, although she had a criminal record. |
| 05/18 | 17 | 1 | 1 | Shooter was a big fan of guns. His father was a fan of the NRA. He took two guns from his father’s closet. He ordered more than 100 rounds of ammunition online despite being underage to do so. The website did not check his age, it only had him check a box where he confirmed that he was old enough. |
| 05/18 | 13 | 1 | 1 | Father and shooter went shooting on gun ranges. The gun was stored in a locked cabinet in the basement next to where the shooter played videogames. One day, he found the keys, opened the safe and got the gun. |
| 08/18 | 12 | 1 | 1 | Shooter was fascinated with guns and looked at guns using the school laptop earlier. He obtained the gun from home, from a locked closet to which he found the key. |
| 02/19 | 16 | 1 | 1 | Shooter took an unsecured handgun out of his parents’ walk-in closet. |
| 04/19 | 22 | 1 | 2 | Shooter bought the gun legally. |
| 05/19 | 16 | 0.7 | 1 | Shooters stole guns from a safe of his partner’s (row below) parents. Shooter had to strike the safe with an axe and pried the door open with a crowbar. |
| 05/19 | 18 | 0.7 | 1 | See row above: The two shooters took the gun from the shooters’ family home. |
| 11/19 | 16 | 0.7 | 1 | Father was an avid hunter who owned many firearms, shooter grew up with guns and was well versed in them. His father had owned handguns in the past but they were lawfully removed. The shooter took a ghost gun from home. It is unclear who assembled the gun, but it was likely his father’s. |
| 05/21 | 12 | NA | NA | Due to the shooters age, data was not made public. |
| 10/21 | 25 | 1 | 2 | Shooter legally purchased the firearm online. |
| 11/21 | 15 | 1 | 1 | His father bought him the gun as a Christmas present four days before shooting on Black Friday. The parents kept their arsenal unlocked. |
| 05/22 | 18 | 1 | 2 | Shooter legally purchased an arsenal right after his 18th birthday. It took him a total of only 41 minutes to get two assault rifles and ammunition with three trips to the store. |
| 10/22 | 19 | 1 | 2 | Shooter tried to buy a gun from a licensed dealer early October, but was denied after an FBI background-check. He then bought the gun used in the shooting later the same month from a private seller. |
| 03/23 | 28 | 1 | 2 | The shooter bought seven firearms at five different stores. The shooter’s parents thought that the shooter had one firearm, but had sold it. |
