# Supplementary material for: “The only friend I had was my gun”: A mixed-methods study of gun culture in school shootings
Source: PLoS One. 2025 Apr 23;20(4):e0322195. doi: 10.1371/journal.pone.0322195 (PMC12017492; doi:10.1371/journal.pone.0322195)
Supplement: Attachment — Submitted filename: PONE review.docx [file pone.0322195.s003.docx]

The author conducted a mixed-method (primarily qualitative) analysis of school mass shootings in the US, under the lens of the role of gun culture for the perpetrators.

I generally agree with the author that this is an important topic. I think the analysis was well done, and the paper is well-written (some grammatical and wording errors and the need to define terminology aside, as noted further down). Some of the findings are interesting and I believe highly relevant in this space.

As laid out below, my major recommendation is to refine the research question and refocus the paper. I think aspects of the paper that the authors talk about and that are important and interesting include:

- Personal meaning mass shooters derive from firearms. This could be contextualized more broadly into literature on the importance of meaning-making as a motivation for human behavior.
- In several places the authors emphasize the role of guns to create a sense of belonging / as a remedy to loneliness. Recent research has shown that many mass shooters are suicidal (see comments below), which I believe points to some dots that could likely be connected here: Thwarted belongingness is a key component in the widely accepted interpersonal theory of suicide (Thomas Joiner). If lack of sense of belonging is common among mass shooters, this could also suggest possible points of intervention, which would potentially be highly relevant.

However, I have some major concerns about the paper.

1) The biggest concern that I have is that the author does not reference relevant work that has been conducted in this space in recent years, and without integrating that work, the manuscript does not seem up to date. Because of those omissions, some of the research questions are not particularly novel since they have been studied and answered before. Foremost, the author does not cite any of Jillian Peterson’s and James Densley’s work, especially their 2021 book:

Peterson, Jillian, and James Densley. *The violence project: How to stop a mass shooting epidemic*. Abrams, 2021.

The author also does not cite any of Jaclyn’s Schildkraut’s work on mass shootings. I am less familiar with the latter’s work, and also have not kept up with Dr. Peterson’s and Dr. Densley’s more recent publications (this may require another review of the literature), but these three authors are among the leading researchers on US mass shootings. Many of the findings in the author’s paper are redundant what these researchers have already described. Therefore, the following statement on page 10 is not accurate:

*To my knowledge, no study to date comparatively analyzed a full sample of rampage school*

*Shootings.*

One of the main research findings presented in the paper, i.e., that most mass shooters, including minors, had easy access to unsafely stored guns, is common knowledge, for example:

https://www.sandyhookpromise.org/blog/gun-violence/facts-about-gun-violence-and-school-shootings/

There is a growing body of literature on the issue of unsafe firearm storage in US, including in households with underage children in recent years, not limited to the mass shootings space. Not citing this body of work seems inappropriate in this study. For example:

Azrael, Deborah, et al. "Firearm storage in gun-owning households with children: results of a 2015 national survey." *Journal of urban health* 95 (2018): 295-304.

Price, James H., and Jagdish Khubchandani. "School firearm violence prevention practices and policies: Functional or folly?." *Violence and Gender* 6.3 (2019): 154-167.

One important previous finding specific to school mass shooters is that many mass shooters are suicidal, which the author also does not comment on.

https://www.theviolenceproject.org/key-findings/

The paper does not mention the cultural relevance of the Columbine shooting as an ‘inspiration’ for copy-cat attacks in school mass shootings (Drs. Peterson and Densley go into that in the above mentioned book if I remember correctly). Another source here:

Cox, John Woodrow. "Children under fire: An American crisis." Ecco, 2021.

2) The author does not contextualize US mass shootings into the larger context of gun violence in the US. While mass shootings, especially in schools, receive most of the media attention, they represent a tiny fraction (<2%) of US gun mortality. That does not mean that mass shootings should not be studied, but I think such a contextualization into the bigger picture of US gun violence is warranted in any paper on US mass shootings.

3) Although the author’s focus is on gun culture as a driving factor, there is no mention of recent work that documents recent changes in demographics among firearm owners: a disproportionate rise in women and persons of color who own firearms, many of them politically left-winged. As the author notes, the vast majority of mass shooters are male, but they are not disproportionately white. Furthermore, gun culture is becoming demographically much mor complicated in recent years. For example:

Miller, Matthew, Wilson Zhang, and Deborah Azrael. "Firearm purchasing during the COVID-19 pandemic: results from the 2021 National Firearms Survey." *Annals of internal medicine* 175.2 (2022): 219-225.

4) The author did not convince me that culture plays a disproportionate role for mass shooters in comparison to US gun owners who never become violent. The immersion into gun culture at young ages is quite common in the US and not unique to mass shooters. In fact, some early immersion gun cultures, e.g., sport shooting / hunting, if combined with emphasis on the importance on gun safety, may be somewhat protective against firearm injuries, for example:

Boine, Claire, et al. "What is gun culture? Cultural variations and trends across the United States." *Humanities and Social Sciences Communications* 7.1 (2020): 1-12.

In the international comparison, the US gun culture is likely a tremendous factor for mass shootings; but within US gun culture, I do not think that the author answers this question that they pose:” Why would the omnipresent and often fairly abstract concept of gun culture translate into practices for the shooters, but not for most people?” I think it is ok not to get to an answer to that question based on their analysis, but I think that should be made explicit in the discussion.

5) The authors use some terminology repeatedly that needs clear definition at the beginning of the paper, including the term ‘culture in action’; who is ‘we’ in “Scholars highlight that we need more research exploring […]. To do so we need to […]”; “the systematic meaning of guns in the US”.

6) The authors frequently jump between present and past tense throughout the manuscript.

7) There are many missing commas / incorrect commas and some wording errors in the text. I recommend running the final manuscript by a native English speaker.

8) There is a large section in the introduction in which the authors mention findings. This should be removed.

9) I would remove this statement from the abstract. “Legislation impacts how firearms are obtained but not the number of shootings”. I do not think the latter part of that statement is a proven fact.

My major recommendations for this paper are to

- Review recent research on the topic that has not been included in this paper.
- Dramatically reduce the Results section on “Easy access and US Rampage School Shootings” since there is a large body of literature in the US on that topic. and instead contextualize the fact that the author confirmed these previous finding in an expanded discussion where that literature is cited. One potentially interesting aspect that could be added here in the discussion: the conviction of the parents of . Holding parents accountable for their children’s access to firearms could have a game-changing impact on US gun storage culture.

https://www.cnn.com/2024/04/09/us/james-jennifer-crumbley-sentencing/index.html

- Refine the research question and refocus the paper. I do think that the author makes some important contributions to the space as mentioned at the top, but this gets lost in presenting findings that are not new and not citing relevant work in this space.
