# Supplementary material for: “The only friend I had was my gun”: A mixed-methods study of gun culture in school shootings
Source: PLoS One. 2025 Apr 23;20(4):e0322195. doi: 10.1371/journal.pone.0322195 (PMC12017492; doi:10.1371/journal.pone.0322195)
Supplement: Attachment — Submitted filename: Response to Reviewers.docx [file pone.0322195.s005.docx]

**Response Letter**

**PLOS ONE**

Manuscript PONE-D-24-36435

“The only friend I had was my gun”: A mixed-methods study of gun culture in school shootings”

I want to express my sincere appreciation to the editor and the reviewer for their careful reading and these thoughtful and constructive comments. I addressed all comments in my revision. In particular I refocus the article and reference more research in the field. In my view, the manuscript greatly benefited from the review and revision. I provide details on each comment below. To highlight where I cited the respective literature, the passages below include the cited references as full citation. In the revised manuscript, the in-text references were already updated to the journal guidelines and changed into numbers. I highlighted all larger changes in yellow in the ‘Revised Manuscript with Track Changes' document.

*Reviewer Comments*

*The author conducted a mixed-method (primarily qualitative) analysis of school mass shootings in the US, under the lens of the role of gun culture for the perpetrators.*

*I generally agree with the author that this is an important topic. I think the analysis was well done, and the paper is well-written (some grammatical and wording errors and the need to define terminology aside, as noted further down). Some of the findings are interesting and I believe highly relevant in this space.*

*As laid out below, my major recommendation is to refine the research question and refocus the paper. I think aspects of the paper that the authors talk about and that are important and interesting include:*

- *Personal meaning mass shooters derive from firearms. This could be contextualized more broadly into literature on the importance of meaning-making as a motivation for human behavior.*
- *In several places the authors emphasize the role of guns to create a sense of belonging / as a remedy to loneliness. Recent research has shown that many mass shooters are suicidal (see comments below), which I believe points to some dots that could likely be connected here: Thwarted belongingness is a key component in the widely accepted interpersonal theory of suicide (Thomas Joiner). If lack of sense of belonging is common among mass shooters, this could also suggest possible points of intervention, which would potentially be highly relevant.*

Response: Thank you for your comments and main recommendations, which I implemented in the revision and which have made the article much stronger, in my view. In the revised manuscript, I emphasize more strongly personal meaning-making and belonging. To achieve this, I restructured the article and reduced the discussion of gun access (see below). I emphasize meaning making in the abstract, when discussing the aims and research questions of the manuscript, and in the literature overview (p.7; p.16; p.19/20). For instance, on page 7, I added: “Meaning-making and belonging are primary human motivations that increase not just psychological well-being, but also resilience and life satisfaction (Lau et al. 2018; see also Frankl, Winslade, and Kushner 2006).”

On p.20, I now highlight: “Older shooters differ from younger shooters, not just because they can legally obtain firearms and because they are more likely to live independently (and are thus less likely to gain access through parents), but also in the symbolic meanings they assign to firearms. While firearms are highly relevant for younger shooters’ belonging and group identity, for older shooters they seem more important for self-identity and well-being. To many older shooters, guns were their only interest, the only way in which they were social and had a hobby. For many, this hobby was a refuge from their anxieties, isolation, and severe mental disorders. Thus, guns positively impacted the older shooters’ well-being and many formed their personal identity around them.”

I also expand on this point on page 22: “But their appeal is amplified for many school shooters: With many of the older shooters feeling desperate and powerless, being marginalized and suffering from mental disorders, findings suggest firearms have a special draw, helping with anxieties and often being the one source of joy, belonging and connection to the outside world (see also Mencken and Froese 2017) . Literature on gun ownership suggests overall gun owners report higher levels of alienation from society and people who feel they are misfits and people who feel economically left behind, feel empowered through guns (Menken & Fraese 2017). Guns thereby tend to create a sense of belonging that most school shooters otherwise lack. Thwarted belongingness, shown to be a vital component in suicides Van Orden et al. 2012; Van Orden, Merrill, and Joiner Jr. 2005), also seems to be a relevant component in many school shooters’ lives (see also Kalish and Kimmel 2010; Newman et al. 2004; The Violence Project 2025).”

On p.26 I summarize point in light of discussed findings and on p. 22 below, I briefly discuss well-being and identity formation in hobbies.

I agree that this lack of belonging could also be a vital angle for intervention and now added this point in the article. P. 27: “Research also needs to reassess prevention measures, since none of the current measures clearly diminish firearm violence in schools (Price and Khubchandani 2019). First, findings underline the role of meaning making and belonging in school shooters’ lives. With belonging being vital for human well-being and life satisfaction (Lau et al. 2018), findings call for more research on prevention measures fostering student belonging and thereby possibly reducing violent behavior overall (see also Jonson 2017; Peterson and Densley 2021).”

To address the grammatical and wording errors mentioned by the reviewer, a native proof reader conducted a thorough proof reading.

*However, I have some major concerns about the paper.*

***1)*** *The biggest concern that I have is that the author does not* ***reference relevant work*** *that has been conducted in this space in recent years, and without integrating that work, the manuscript does not seem up to date. Because of those omissions, some of the research questions are not particularly novel since they have been studied and answered before. Foremost, the author does not cite any of Jillian Peterson’s and James Densley’s work, especially their 2021 book: Peterson, Jillian, and James Densley. The violence project: How to stop a mass shooting epidemic. Abrams, 2021.*

*The author also does not cite any of Jaclyn’s Schildkraut’s work on mass shootings. I am less familiar with the latter’s work, and also have not kept up with Dr. Peterson’s and Dr. Densley’s more recent publications (this may require another review of the literature), but these three authors are among the leading researchers on US mass shootings. Many of the findings in the author’s paper are redundant what these researchers have already described. Therefore, the following statement on page 10 is not accurate: To my knowledge, no study to date comparatively analyzed a full sample of rampage school Shootings.*

Thank you for this comment. I conducted another thorough review of the literature and now discuss Peterson and Desley’s work, as well as Schildkraut’s work. I added Peterson & Densley’s research among other instances on page 5, where I discuss gun related deaths during the Covid pandemic, and on page 8 when contextualizing firearm morbidity and school shootings (see also reply to your comment below). I also more closely discuss the work by Schildkraut and added further works in this field, when contextualizing school shootings on page 8 and 9.

p.5: “During the Covid-19 pandemic gun purchases further increased by an estimated 64% (EveryTown 2021; see also Peterson and Densley 2021).”

p.7/8: “Although rampage school shootings inherently gain massive media attention, only 2% of youth firearm homicides occur at schools and only a fraction of these are rampage school shootings (Price and Khubchandani 2019:163). Mass shootings, meaning events in which four or more victims are killed across a venue (workplace, school, or other, Peterson and Densley 2021:3), only represent 1% of gun violence in America; and among these only 25.1% occur at school (Brady Campaign 2025; Schildkraut 2021).”

See also page 8: “Studies on various types of mass shootings underline the ease with which shooters obtain firearms and the strong affection many hold for their guns (Peterson and Densley 2021; Schildkraut 2021).”

I also discuss Densley and Peterson’s work, when I discuss social belonging on page 27 (see my response to your first comment above), and Schildkraut’s work on safe storage on page 28 (see my response to your comment on safe storage below).

In addition, I highlight more clearly the difference between mass shootings and rampage school shootings and clarify that a lot of the discussed work studies mass shootings, while my study looks at a full sample of school shootings – meaning all instances of random shooting sprees at schools, regardless of the number of victims. I realize that this distinction was not clear enough in the earlier version and now highlight it in great detail throughout the text.

E.g., on page 4 I specify: “I analyze a full sample of school shootings; incidents that result in mass shootings (four or more killed) as well as attacks where fewer people or no one was killed.”

In the methods section, I highlight that a study on all types of school shootings (including mass shootings and those with less than four victims) can add a vital dimension to this field of study:

p. 9: “To do so, I study gun culture across a full sample of school shootings, i.e., all random active shooter attacks at school. The sample thereby includes mass shootings, as well as shootings with fewer victims and incidents during which no one was killed or injured. Mass shootings (four or more killed) cover the most gruesome and most “successful” cases from the perspective of the perpetrator, but school shootings with less victims are much more common than those resulting in mass murder (Author 2024). To understand the full range of the phenomenon, research needs to include every case where a current or former student went to their school to randomly fire at people, even if they were stopped shortly after. Research points out that attacks often tend to fail due to situational dynamics (Author 2024) and that the speed of medical interventions (e.g., distance of the school to the next hospital) influences how many victims die and whether an attack results in a mass shooting. Research also shows all types of school shooting attacks lead to horrors for survivors and trauma across the US school system, which further increases the relevance of including ultimately failed attempts (Cox 2021).”

I now emphasize this point throughout the rest of the manuscript, while highlighting how my findings relate to research on mass shootings and more closely tying my findings in with findings by Peterson and Densley, Schildkraut, and others.

*One of the main research findings presented in the paper, i.e., that most mass shooters, including minors, had* ***easy access to unsafely stored guns****, is common knowledge, for example:*

*https://www.sandyhookpromise.org/blog/gun-violence/facts-about-gun-violence-and-school-shootings/*

*There is a growing body of literature on the issue of unsafe firearm storage in US, including in households with underage children in recent years, not limited to the mass shootings space. Not citing this body of work seems inappropriate in this study. For example:*

*Azrael, Deborah, et al. "Firearm storage in gun-owning households with children: results of a 2015 national survey." Journal of urban health 95 (2018): 295-304.*

*Price, James H., and Jagdish Khubchandani. "School firearm violence prevention practices and policies: Functional or folly?." Violence and Gender 6.3 (2019): 154-167.*

In line with this comment, your first and last comment of this review, I now switched the main focus of the article to meaning making around firearms and focus less on gun access (see below). In the passages where I still discuss access, I added the findings summarized by the Sandy Hook Promise, as well as the research on firearm storage by Azrael, Price, and others.

On page 17, when discussing findings, I added: “These findings are in line with research showing that in all types of school violence, parents are the most common source for firearms (Hovey et al. 2017) and 7% of children in the US live in homes where a firearm is stored unlocked and loaded (Azrael et al. 2018:295; see also Parikh et al. 2017).”

In the Conclusion (p.28) I now discuss safe storage more extensively: “Further, findings suggest that to prevent school shootings, not only must the prevalence of guns and legal access for potential shooters over 18 be considered, but also easy unguarded access for children at home. More research is needed on how guns can be stored safely away from children and young adults. With research indicating that gun ownership is generally increasing, including among previously underrepresented groups, the relevance of safe storage will further increase. Between 2002 and 2015, the number of children living in homes with at least one unlocked and loaded firearm has increased from about 1.6 million to 4.6 million (Azrael et al. 2018:298). Research highlights that safe storage practices – storing firearms and ammunition separately – can decrease the risk of firearm deaths among children (Miller, Azrael, and Hemenway 2002; Schildkraut 2021; see also Sandy Hook Promise 2025). Findings suggest not having a firearm at home, or if a firearm is stored, storing it safely, may not just help prevent accidents and suicides with firearms at home, but also planned attacks, like school shootings.”

I also discuss storage when discussing the role of parents in prevention efforts on page 28/29 (see your last comment). I also discuss the work by Price when discussing firearm deaths, contextualizing school shootings, and discussing prevention on page 6, 7, 27, and research by the Sandy Hook Promise on page 28.

*One important previous finding specific to school mass shooters is that* ***many mass shooters are suicidal****, which the author also does not comment on. https://www.theviolenceproject.org/key-findings/*

*The paper does not mention the cultural relevance of the Columbine shooting as an ‘inspiration’ for copy-cat attacks in school mass shootings (Drs. Peterson and Densley go into that in the above mentioned book if I remember correctly). Another source here: Cox, John Woodrow. "Children under fire: An American crisis." Ecco, 2021.*

I now discuss research in this field in more detail. For instance, on page 8: “Scholars highlight that many school shooters, especially those who kill many victims, are suicidal, many suffering from childhood trauma, marginalization, and mental disorders (Newman et al. 2004; Peterson and Densley 2021; Rocque 2012). Further, many shooters show distinct masculinity scripts (Borgogna, McDermott, and Brasil 2022; Kalish and Kimmel 2010) and are inspired by previous shooters (Cox 2021; Towers et al. 2015).”

I also reference literature on suicides on page 22, when discussing thwarted belongingness (see above): “Thwarted belongingness, shown to be a vital component in suicides (Van Orden et al. 2012; Van Orden, Merrill, and Joiner Jr. 2005), thereby also seems to be a relevant component in many school shooters’ lives (see also Kalish and Kimmel 2010; Newman et al. 2004; The Violence Project 2025).”

Throughout the article, I cite more publications by The Violence Project and Cox, e.g., in the second sentence of the introduction (p.3), or when discussing the trauma resulting from school shootings (p.9), see also p. 5, 7, 8, p. 22.

*2) The author does not* ***contextualize US mass shootings into the larger context of gun violence*** *in the US. While mass shootings, especially in schools, receive most of the media attention, they represent a tiny fraction (<2%) of US gun mortality. That does not mean that mass shootings should not be studied, but I think such a contextualization into the bigger picture of US gun violence is warranted in any paper on US mass shootings.*

I agree that this contextualization is important and added it to the revised manuscript.

p. 6: “Research on gun related deaths shows that the US also has some of the highest rates of firearm deaths among high-income countries, while firearm morbidity among young people is on the rise (Aneja, Donohue, and Zhang 2012; Hemenway 2006; Malcolm 2004; Siegel, Ross, and III 2013). In 2021, guns accounted for nearly 19% of deaths among American children and teens – surpassing car accidents, cancer, and all other causes of death among those 18 and younger (Choi 2023; Grinshteyn and Hemenway 2016; see also Price and Khubchandani 2019).”

p. 7/8: “Although rampage school shootings inherently gain massive media attention, only 2% of youth firearm homicides occur at schools and only a fraction of these are rampage school shootings (Price and Khubchandani 2019:163). Mass shootings, meaning events in which four or more victims are killed across a venue (Peterson and Densley 2021:3), only represent 1% of gun violence in America; and among these only 25.1% occur at school (Brady Campaign 2025; Schildkraut 2021).”

*3) Although the author’s focus is on gun culture as a driving factor, there is no mention of recent work that documents* ***recent changes in demographics among firearm owners****: a disproportionate rise in women and persons of color who own firearms, many of them politically left-winged. As the author notes, the vast majority of mass shooters are male, but they are not disproportionately white. Furthermore, gun culture is becoming demographically much mor complicated in recent years. For example: Miller, Matthew, Wilson Zhang, and Deborah Azrael. "Firearm purchasing during the COVID-19 pandemic: results from the 2021 National Firearms Survey." Annals of internal medicine 175.2 (2022): 219-225.*

Thank you for this comment. I now added this finding in the article (p.6): “However, between 2019 and 2021 studies find a disproportionate increase in previously underrepresented groups becoming gun owners, including women and people of color (Miller, Zhang, and Azrael 2022).”

I also emphasize this point in the Conclusion (p. 28): “With research indicating that gun ownership is generally increasing, including among previously underrepresented groups, the relevance of safe storage will further increase.”

*4) The author did not convince me that* ***culture plays a disproportionate role*** *for mass shooters in comparison to US gun owners who never become violent. The immersion into gun culture at young ages is quite common in the US and not unique to mass shooters. In fact, some early immersion gun cultures, e.g., sport shooting / hunting, if combined with emphasis on the importance on gun safety, may be somewhat protective against firearm injuries, for example: Boine, Claire, et al. "What is gun culture? Cultural variations and trends across the United States." Humanities and Social Sciences Communications 7.1 (2020): 1-12.*

*In the international comparison, the US gun culture is likely a tremendous factor for mass shootings; but within US gun culture, I do not think that the author answers this question that they pose:” Why would the omnipresent and often fairly abstract concept of gun culture translate into practices for the shooters, but not for most people?” I think it is ok not to get to an answer to that question based on their analysis, but I think that should be made explicit in the discussion.*

I fully agree with your point and now added a longer reflection in the discussion to highlight this distinction. (The article by Boine et al does not seem to discuss whether early immersion can prevent firearm injuries, but I discuss the article by Boine et al on page 7 when discussing variations and trends in US gun culture. When discussing immersion and the risk of accidents, suicides and homicides in young children I discuss research by Miller et al (page 6, see also p. 28).)

On the role of gun culture, I re-phrased the key interest of the article on p.4: “We need more insights into whether gun culture actually matters for school shooters, and whether school shooters show indicators of culture in action that led to distinct meanings or practices around guns.”

In the Conclusion (p.27), I added: “The finding that school shooters come from a distinct cultural pocket in the US, a leisure gun culture that is otherwise declining, narrows down the group of possible perpetrators. Yet, only a very small sub-group of Americans with easy gun access go on a rampage. Therefore, a limitation of this study – and others in the field – is the lack of negative comparison cases: More research is needed on students with the same context factors such as marginalization or family trauma, as well as easy gun access, who never commit a school shooting. Moreover, future research should examine whether this gun culture in action only describes a shooter profile, or whether it also holds relevance for the shooters’ decision to commit a shooting. More insights are needed whether those who contemplate committing a shooting but do not have easy access to firearms decide against a shooting, or if easy access gives shooters the idea to commit a shooting.”

*5) The authors use some terminology repeatedly that needs clear definition at the beginning of the paper, including the term ‘culture in action’; who is ‘we’ in “Scholars highlight that we need more research exploring […]. To do so we need to […]”; “the systematic meaning of guns in the US”.*

Thank you for pointing this out. I now define culture in action on page 3: “Swidler (1986:273) describes culture as “symbolic vehicles of meaning, including beliefs, ritual practices, art forms, and ceremonies, as well as informal cultural practices such as language, gossip, stories, and rituals of daily life.” Her concept of “culture in action” understands culture as more than merely a set of static values. It emphasizes that culture functions as a flexible toolkit of symbols, practices, and strategies that individuals draw on to navigate and shape their actions in specific contexts. Importantly, this understanding sees culture as dynamic and as constantly shaped through social interactions with others.”

In addition, I rephrased all sentences previously including “we” (p. 7; p. 8; p. 10).

*6) The authors frequently jump between* ***present and past tense*** *throughout the manuscript.*

*7) There are many* ***missing commas*** */ incorrect commas and some wording errors in the text. I recommend running the final manuscript by a native English speaker.*

Thank you for pointing this out. I now use the same tense throughout the manuscript and the manuscript was checked again by a native proof reader to avoid incorrect commas and other spelling mistakes.

*8) There is a large section in the introduction in which the authors mention findings. This* ***should be removed****.*

*9) I would* ***remove this statement from the abstract****. “Legislation impacts how firearms are obtained but not the number of shootings”. I do not think the latter part of that statement is a proven fact.*

Thank you for these comments. I removed this entire section on page 13 and I removed the sentence on legislation from the abstract.

***My major recommendations*** *for this paper are to*

- *Review recent research on the topic that has not been included in this paper.*
- *Dramatically reduce the Results section on “Easy access and US Rampage School Shootings” since there is a large body of literature in the US on that topic and instead contextualize the fact that the author confirmed these previous finding in an expanded discussion where that literature is cited. One potentially interesting aspect that could be added here in the discussion: the conviction of the parents of . Holding parents accountable for their children’s access to firearms could have a game-changing impact on US gun storage culture.* [*https://www.cnn.com/2024/04/09/us/james-jennifer-crumbley-sentencing/index.html*](https://www.cnn.com/2024/04/09/us/james-jennifer-crumbley-sentencing/index.html)
- *Refine the research question and refocus the paper. I do think that the author makes some important contributions to the space as mentioned at the top, but this gets lost in presenting findings that are not new and not citing relevant work in this space.*

Thank you for highlighting these points. I refocused the paper and now cite more relevant work. I conducted another review of the literature and added more recent research in the field, while highlighting the differences between mass shootings and school shootings (see above).

I greatly reduced the results section “Easy access and US Rampage School Shootings” and I now changed the structure of the paper to emphasize my findings on the meaning making of guns. The first two sections now discuss meaning making (p.13-22), while a third, and strongly shortened, section briefly discusses gun access (p.23-25).

I highlight this new structure when discussing the paper’s questions on page 11, as well as when discussing its structure on page 12: “In the following sections, findings from my in-depth case analyses and cross case comparisons will carve out meanings and practices around firearms among school shooters. Although specific meanings differ in younger and older shooters, for the majority of shooters (for details see Supporting Information S2), guns played a vital role in their lives prior to the shooting. Relying on descriptive statistics, a last section will discuss how culture in action shapes practices given that *all* US school shooters show easy gun access.”

I agree with you on the relevance of holding parents accountable and expand on this in the conclusion, where I discuss practical implications of the findings. P.28/29 (see also my reply to your comment on safe storage above): “Third, findings underline the role of parents or other adults in gun access. Among all rampage school shooters under 18 – the minimum legal age to purchase a firearm in most US States – none showed difficulties obtaining a firearm. This may indicate that those who face difficulty in obtaining a firearm simply do not commit a school shooting. Thereby, findings emphasize that avoiding access through adults, especially parents, is vital for prevention. 2024 marked the first time in history that a lawsuit held the parents of a school shooter accountable. The parents of 15-year-old Billy, who attacked his Michigan school, were convicted of manslaughter and sentenced to 10 to 15 years in prison due to their son’s “unfettered access to a gun or guns as well as ammunition in [their] home” and since they “glorified the use and possession of these weapons,” according to the judge (Sanchez 2024). These two aspects mentioned by the judge directly correspond to meaning making around, alongside access to, firearms, as discussed in this article. In light of these findings, helping parents understand what safe storage means and holding them accountable for unsafe storage are worth exploring as avenues for effective prevention. However, a positive effect of longer prison sentences for parents, as in Billy's case, can be called into question given research showing that longer prison sentences do not affect crime deterrence or reoffending (see Mauer 2018; Turner 2018).
